# Triple-renewable energy system for electricity production and water desalination

**DOI:** 10.1007/s11356-022-22547-2

**Published:** 2022-08-29

**Authors:** Emad Abdelsalam, Fares Almomani, Hamza Alnawafah, Radi Alrashed

**Affiliations:** 1 Energy Engineering Department, Al Hussein Technical University, Amman, 11831 Jordan; 2 Department of Chemical Engineering, College of Engineering, Qatar University, Doha, Qatar

**Keywords:** Solar energy, Solar chimney, Cooling tower, Photovoltaic, Water distillation

## Abstract

This work presents a novel triple-renewable energy system (TRES) that is based on integrating the photovoltaic panels (PVPs), conventional solar chimney (CSC), and cooling tower (CT) in one structure. The ultimate objective of the proposed TRES system is to produce electrical power (P_elc_), desalinated water (D_w_), and if required cooling utilities. The components of the system include a chimney tower, collector, base, PVPs, water pool, bi-directional turbine, and water sprinklers. The TRES system can be operated as CSC during the daytime and CT at night providing 24-h operation. The PVPs were integrated within the structure to increase the P_elc_ production and enhance the process performance by heating the air inside the system. The TRES structure increased the efficiency to 0.860% in comparison with the CSC (0.313%). The annual P_elc_ production from the TRES system was found to be 792 MWh compared with only 380 MWh generated by the CSC achieving 2.1 folds overall improvement. The CSC-PV and CT contributed to 47% (494 MWh) and 24% (253 MWh) of the P_elc_ production, respectively. The annual D_w_ production was found to be 1.2-fold higher (163,142 tons) higher than the CSC (139,443 tons). The newly developed TRES system offers a great potential to produce P_elc_ and D_w_ and save fossil fuel consumption while reducing the emissions of greenhouse gasses (GHGs) to the atmosphere.

**Supplementary Information:**

The online version contains supplementary material available at 10.1007/s11356-022-22547-2.

## Introduction

The worldwide need for energy rises resulting in an energy crisis. The energy derived from fossil fuels is unsustainable, contributes to environmental pollution, and increases the emission of greenhouse gasses (GHGs) into the atmosphere (Hasanuzzaman et al. 2016). As a result, there is a growing demand for environmentally friendly and sustainable alternative energy sources that would offer zero pollutants emissions and support off-grid industrial areas, healthcare, and transportation systems (Attari et al. 2021; Rofiqul Islam et al. 2008; Saha et al. 2022; Sharifi et al. 2021). Aside from energy difficulties and demand issues, water scarcity is a global concern that is expected to worsen over time (Hoekstra et al. 2012; Jia et al. 2020). To that end, considerable effort is being expended in developing novel technologies that will provide a long-term solution to the energy and water challenges.

Solar energy is one of the most importantly clean and free renewable energy sources, which could satisfy the global electrical or thermal energy demand (Kannan and Vakeesan 2016, Sukhatme and Nayak 2017). In general, solar energy is based on harnessing the sun’s energy to generate hot water or electricity via solar photovoltaic panels (PVPs) and concentrating solar power (CSP) systems (Al-Smairan et al. 2020; Purnachandrakumar et al. 2022; Sivakumar et al. 2021). Many solar systems have been installed around the world during the previous few decades, demonstrating the viability of these technologies (Rofiqul Islam et al. 2008; Rourke et al. 2009). However, these systems are made of variety of chemicals and substances that could increase the total cost and may harm the environment. As a result, research efforts have shifted to inventing new technologies that are simple in design, use fewer (or no) chemicals, and require less maintenance.

Seawater desalination, on the other hand, is considered one of the most promising alternatives for providing clean water (Alkaisi et al. 2017; Srimuk et al. 2020). Water desalination using membrane and thermal techniques has been successfully implemented in a variety of commercial projects around the world (Alhaj and Al-Ghamdi 2019). However, the massive energy demand and environmental concerns associated with the emissions of GHGs during water desalination processes are of significant concern (Cornejo et al. 2014). Therefore, deploying a new water desalination technique that uses little/no energy and has a low influence on the environment will make desalination operations more sustainable and cost-effective.

Solar chimney (SC) technology is regarded as an outstanding renewable and sustainable power generating plant due to its simple design (no mechanical parts, low maintenance, no electrical consumption), very low environmental impact (no global warming effects or pollution), and dual-action as it can be used for both heating and cooling (Al-Smairan et al. 2020, Infield and Freris 2020, Natarajan et al. 2022, Panwar et al. 2011). The first design of the conventional solar chimney (CSC) concept for the generation of electrical power (P_elc_) was first proposed in Spain in the late 1970s and operated in 1982 (Kasaeian et al. 2017; Schlaich 1981; Zhou et al. 2010). The design consists of a transparent glass collector, wind turbine, and chimney. The intercepted solar radiation warms the collector’s surface and heats the air at the entrance of the CSC, and this convective heat transfer effect creates a boundary that develops air movement within the structure. The acceleration of air in the chimney operates the turbine and generates P_elc_.

Since the discovery of the CSC, efforts were focused on examining various designs modifications or optimizing the existing structure to maximize productivity and enhance performance. The design changes proposed to the structure of the CSC system focused on reducing the chimney height (H_c_), diameter (D_c_), and collector area (A_c_) without affecting the performance. The low thermal efficiency, high levelized cost of energy, and the requirements for the large land area were also challenges facing the large-scale application of this technology. Other efforts were focused on integrating the CSC with other technologies to increase P_elc_ generation, produce other products, increase thermal efficiency, and lower the capital and operating costs. The directions of these efforts varied between experimental approach (Jing et al. 2015; Saifi et al. 2012), simulation approach (Kasaeian et al. 2014; Sangi et al. 2011), process optimization (Pretorius and Kröger 2006), and numerical calculations (Fasel et al. 2013; Pastohr et al. 2004). Schlaich et al. (2004) and Tingzhen et al. (2008) worked on optimization of the CSC structure (H_c_, D_c_, and A_c_) to maximize P_elc_. Other designed changes include adding a ventilation system to the roof of the CSC (Fluri and Von Backström 2008, Mathur et al. 2006, Okoye and Atikol 2014), using multi turbo generators (Fluri and Von Backström 2008), changing the absorber materials of the collector (Abdelmohimen and Algarni 2018, Abdelsalam et al. n.d., Zandian and Ashjaee 2013, Zuo et al. 2012) and installing insulating material at the surface of the collector (Zuo et al. 2012).

Combining the CSC with other systems was found as a promising alternative to improve the system’s productivity and performance. Recently, different researchers have discussed the inclusion of a seawater basin within the collector of the CSC to promote water desalination (Abdelsalam et al. 2020; Kiwan et al. 2020, Kiwan and Salam 2018). In such a design, the water absorbs solar heat, evaporates, and then condenses on the inner walls of the chimney producing D_W_. Kiwan and Salam (2018) suggested that integrating PVPs and adding a seawater pool at the base would help to achieve PV cooling, generate extra P_elc_, and increase the D_W_ production. Later on, it was confirmed that this combination not only increases the D_w_ and P_elc_ production but also allows the PVPs-CSC system to cool the PVPs for better performance (Kiwan et al. 2020). The combination of the PVPs-CSC increased the utilization efficiency up to 4.37% compared with 0.51% for CSC. The performance and feasibility of the CSC in different geographical areas were also assisted and discussed in our previous work (Abdelsalam et al. 2021a). Zuo et al. (2020) increased the utilization efficiency of the CSC to 15.4% by adding to the top of the CSC a wind turbine to generate extra P_elc_ from the updraft exiting wind. Rashidi et al. (2021) and Aliaga et al. (2021) suggested alternatives to increasing the overall efficiency and lowering the capital costs. In the first work, phase change materials (PCMs) were added to the CSC to enhance the ventilation process and maximize the operation time at a high temperature. The second work used computational fluid dynamics (CFD) code COMSOL to simulate the CSC and determine the optimal conditions and dimensions for maximum power output. The optimized design achieved a higher power density compared with CSC. Recently, we have developed two revolutionary designs of the SCPP. The first one consisted of two co-centric towers, where the inner tower operated as conventional SCC, and the outer tower was divided into ten cooling towers (Abdelsalam et al. 2021b). The second design was denoted as hybrid CSC by adding water sprinklers at the top of the chimney. The system can be operated either as CSC or as CT offering 24-h of P_elc_ and D_w_ generation (Abdelsalam et al. 2021b).

The aforementioned system analysis and literature review revealed that although the stand-alone CSC system could be employed to produce P_elc_ from the solar irradiations, the system suffers from high cost, low thermal efficiency and P_elc_ yield. In addition, the CSC without modifications cannot produce desalinated water (D_w_). Therefore, there is a big need to modify the existing design to improve its feasibility and enhance its efficiency and performance while reducing the required land area and cost. Combining the P_elc_ with additional products (e.g., D_w_) would also promote the system’s feasibility. Consequently, this work demonstrates for the first time the development of a new and unique design of a triple-renewable energy system (TRES) to produce electrical power (P_elc_) and desalinated water (D_w_). The TRES consists of an integrated CSC, PVPs, and a CT in one compact structure, which is considered a third-generation development of the CSC. The new design offers a compact CSC structure with high efficiency, low land requirement, and reduced cost. The operation and performance analysis of the proposed TRES system was analyzed using a simulation process and mathematical modeling under steady-state conditions and using local weather data. The proposed TRES demonstrates the benefit of integrating CT with CSC to extend the operating hours to 24/7, provide a sustainable energy system, and lower GHG emissions. In addition to generating P_elc_ and D_w_, the new design can provide cooling utilities to any nearby industries. The sustainable nature of this structure makes it attractive to be used in/off-grid communities. The system has a very low number of mechanical parts, therefore maintenance cost is minimum. The PV panels are attached to the rim of the collector to utilize the heat generated from these panels to warm the air at the entrance of the collector, thereby enhancing the P_elc_ generation and process efficiency.

## Materials and methods

### Description of the triple-renewable energy system (TRES)

**Fig. 1:**
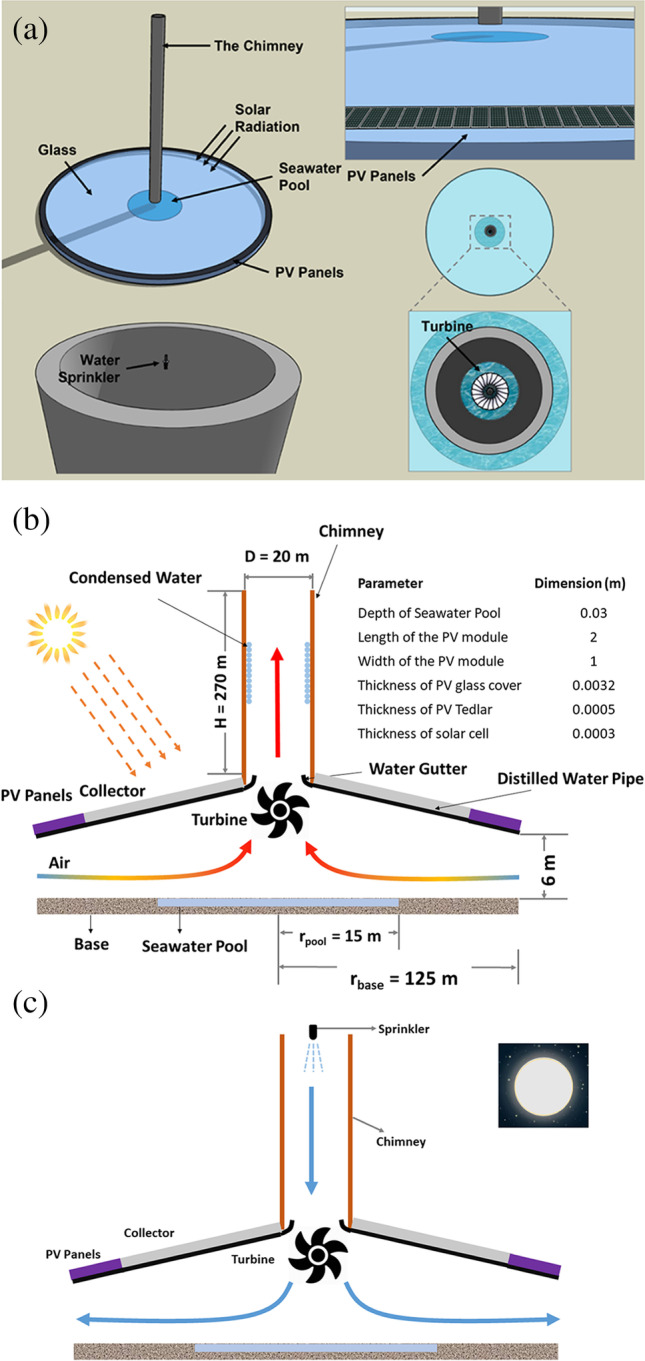
The proposed structure of the TRES: **a** 3D view, **b** 2D view running as SCPP, and **c** is 2D view running as a cooling tower

The structure of the TRES is presented in Fig. 1. The 3D presentation of the proposed structure (Fig. 1a) consists of a collector, water pool, chimney, base, PV panels, a bi-directional turbine, and water sprinklers. The PV panels were attached to the collector’s perimeter. A cross-sectional view of the TRES structure, physical dimensions, and system components are presented in Fig. 1b. The system can be operated as CSC or CT. The direction of the airflow inside the system during the operation as CSC or as CT is indicated by the colored arrow in Fig. 1b and c, respectively. In general, the CSC operates during the day, while the CT mode operated at night. Based on mass and energy balance, the TRES structure consists of four sectors (PV, air heating, air humidification, and the chimney). The PV sector includes the external PVPs that are located at the perimeter of the collector. The air heating sector begins from the rim of the PVPs to the first side of the water pool. The water evaporation sector is bounded by the water basin at the bottom of the structure. Lastly, the column where the air–water moisture travels upward/downward represents the chimney sector.

The collector is considered the main component of the TRES system. The roof of the collector is covered with glass, which converts the intercepted solar irradiation (S_irr_) into thermal energy. As a result, the temperature of the air under the collector increase. Both beam and diffuse solar radiation can be absorbed by the collector. As the temperature of the PVPs increased substantially due to solar irradiation, they were placed at the collector’s entrance to be cooled by the incoming ambient and contributing to inlet air heating. As a result, the TRES structure could be operated in both clear and cloudy conditions. The roof’s height rises gradually from the outer perimeter to the center. Therefore, most of the incident solar energy passes through the roof transparent section, where some are absorbed by the base and others are either reflected or absorbed in the system environment. This also will contribute to the heating of the air at the entrance of the structure. Furthermore, the soil or concrete underlying the roof acts as a short-term thermal storage medium, storing, and discharging thermal energy throughout the day and night, respectively. This would help TRES in overcoming solar radiation’s intermittency.

The bi-directional turbine and the chimney are the other major components of the TRES, which is placed in the center of the collector. The differential temperature created between the inside and ambient air develops the chimney effect causing the air to flow upward the chimney. The airflow rotates the turbine and provides the mechanical energy to produce electrical energy. The turbine of the SC is similar in design to a standard wind turbine, and it was designed to be stable under all weather conditions and to resist the change in temperatures and pressure. Hot air traveling over the seawater pool evaporates water and increases the air humidity. As the hot and humid air flows upward the chimney, its temperature decreases allowing for water condensation on the chimney’s inner walls. The condensed water is captured by the water gutters and is then moved through the water pipes outside the system for storage. The CT is the last major component of the TRES. A mist of water is sprayed from the water sprinklers (located at the top of the tower) to quickly absorbed the dry air. Consequently, the air temperature decrease and it becomes denser forcing it to flow downwards and exchange energy with the turbine, resulting in extra energy production before exiting the system. The usage of a bi-directional turbine in the proposed TRES is a significant design parameter that can operate in both directions (clockwise or counterclockwise) depending on the system operating mode. Depending on the weather circumstances, the system can be operated in a variety of modes. Generally, the CSC and CT are built and operated separately. However, as the CSC can only operate during the day and the CT at night the proposed TRES contain both and can be operated 24 h to increase the efficiency and P_elc_ generation.

### Mathematical model

**Fig. 2:**
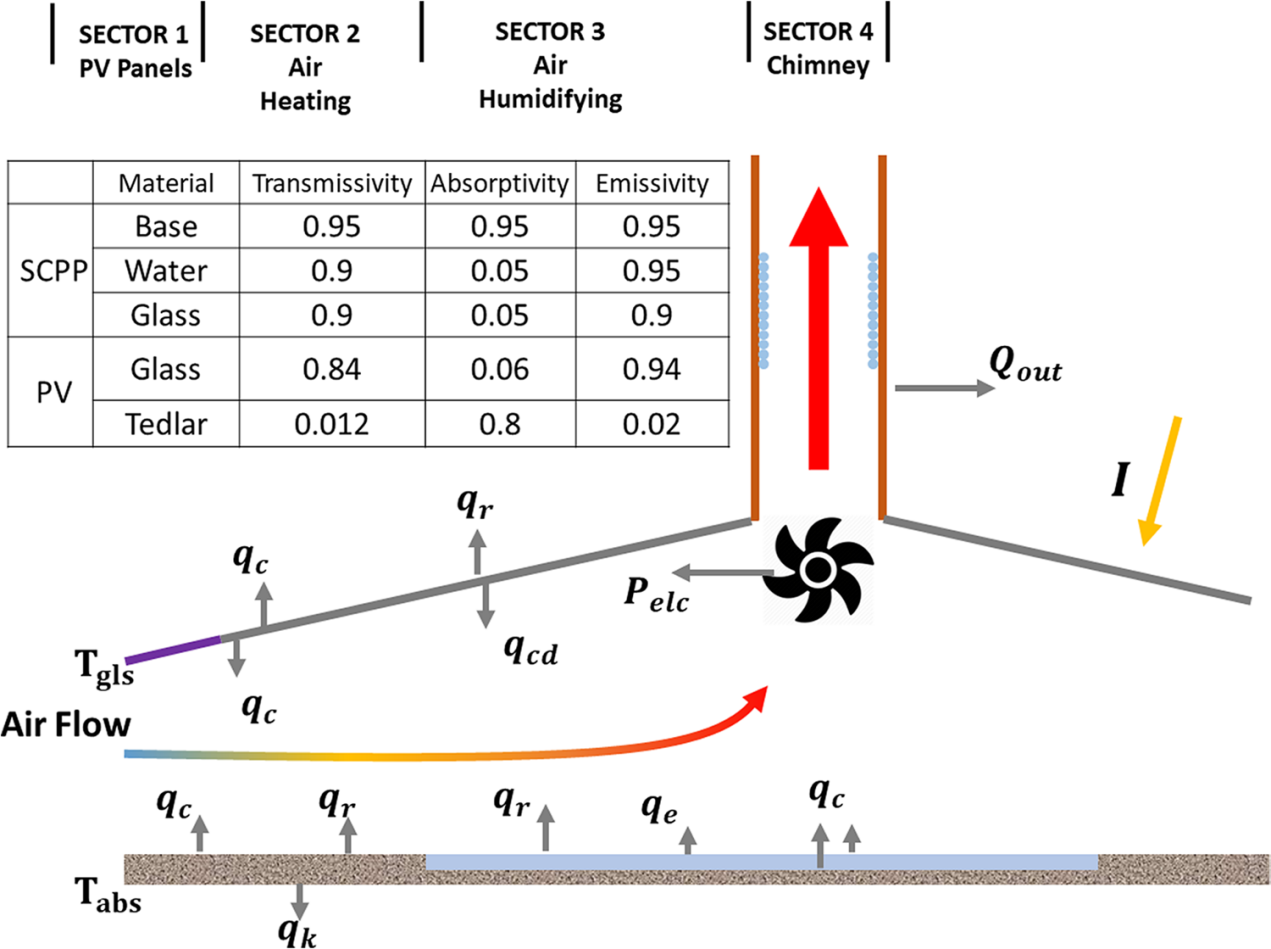
Energy and mass balance representation in the four sectors of the TRES

The performance of the proposed TRES system was analyzed using mathematical modeling. The model was developed based on mass and energy balance over the four sectors of the TRES structure with characteristics presented in Fig. 2. The simulation process was performed under steady-state conditions, assuming no friction, symmetrical airflow, and fixed seawater level.

**Fig. 3:**
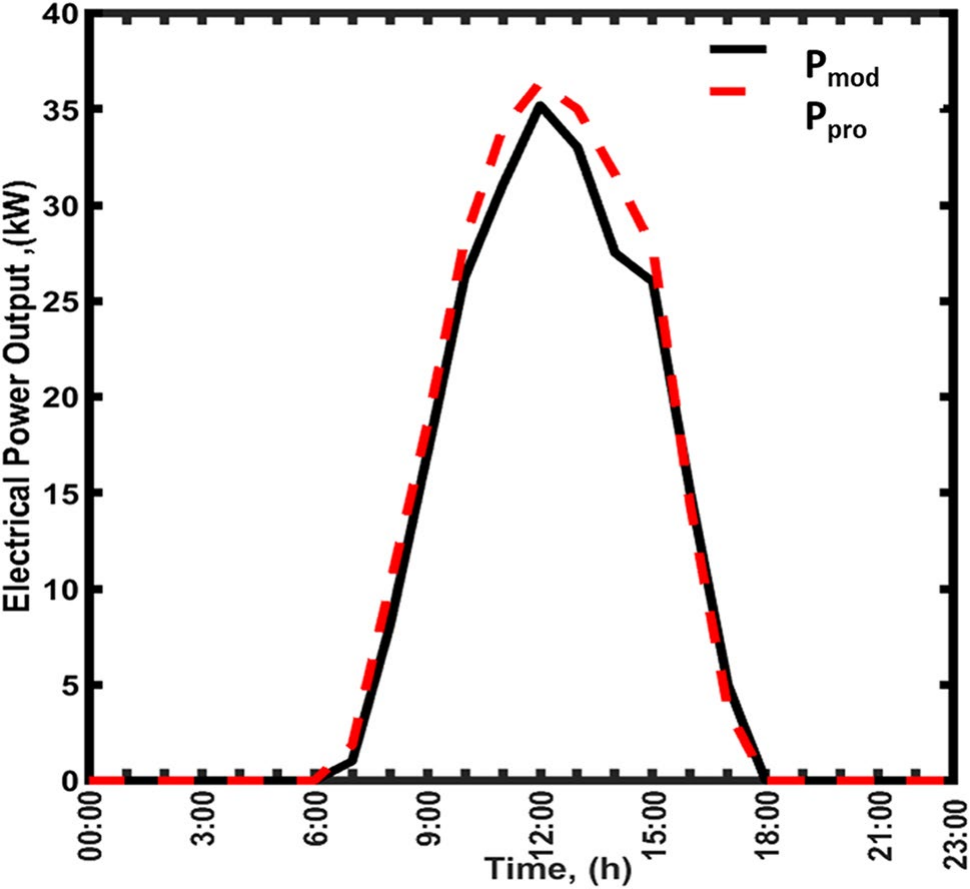
Validation of the proposed model against the prototype results

The mathematical model consists of a set of equations representing the four sectors of the TRES. The set of equations is presented in detail in the supplementary data. The equations were solved numerically using MATLAB-built genetic iterative technique (MATLAB R2013a) to determine the temperature at all sectors within the structure. At any time, the program reads the local weather data from the input file and processes them through the model using the TRES dimensions (see Fig. 1) and thermal-radiative characteristics (Fig. 2). The mathematical model for the CSC without the PV, water pool, and CT was validated against the results reported by Haaf et al. (1983), which is considered the best optimal baseline for the CSC prototype. Figure 3 shows the 24-h P_elc_ generation profile obtained from the proposed model (P_mod_) against the power from the CSCprototype (P_pro_) operated by Haaf et al. The values of P_mod_ and P_pro_ are identical suggesting a strong correlation between the prototype and the model. Statistical analysis shows that 98% of the power values are within ± 3% of the line of standard deviation confirming that the developed model accurately follows the real prototype results. The residuals between the P_mod_ and P_pro_ are scattered around the horizontal zero line with an error in the range of − 0.25 to 0.25 confirming a very small variation between them.

## Results and discussion

The performance of the TRES system is dependent on the weather conditions, specifically the solar radiation (S_irr_), temperature (T), relative humidity (%H_u)_, and wind speed (W_s_). Hence, to assess the structural performance for a whole year, the profiles of the mentioned weather conditions need to be examined in detail. In this part, the daily and yearly weather parameters will be shown.

### Weather profile

**Fig. 4:**
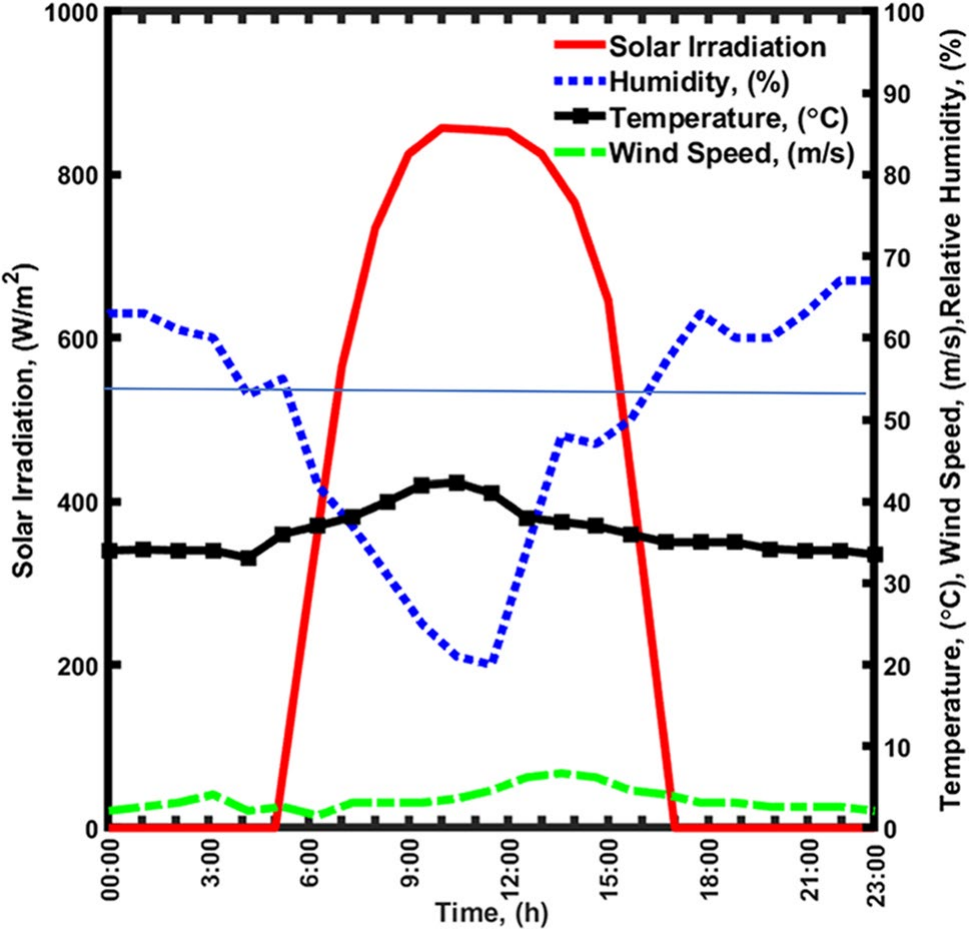
Twenty-four-hour profile of weather data (S_irr_, %H_u_, T, and W_s_) in August 30th, 2021

The hourly weather data was obtained from the local weather station in Doha, Qatar. An example of a 24-h weather profile on August 30th is shown in Fig. 4. The S_irr_ starts at sunrise at approximately 5:00 and increases to reach a peak value of 857 W/m^2^ at noontime. Then, it decreases to become zero at sunset (nearly 17:00). The recorded data on that day shows an average S_irr_ of 314.29 W/m^2^. The %H_u_ profile exhibited an opposite behavior, where the values are in the range of 63 to 52% during the night (i.e., from 3:00 to 6:00). There was a sharp decrease during the day (from 6:00 to 15:00) reaching a minimum value of 20% at the noontime and then returning to increase and reaching a maximum value of 68% at the beginning of the day. The T values are changing in the range of 30 to 40 °C, with a peak value of 40 °C at noontime. Lastly, a variety of W_s_ could be observed between 2.1 and 6.7 m/s.

**Fig. 5:**
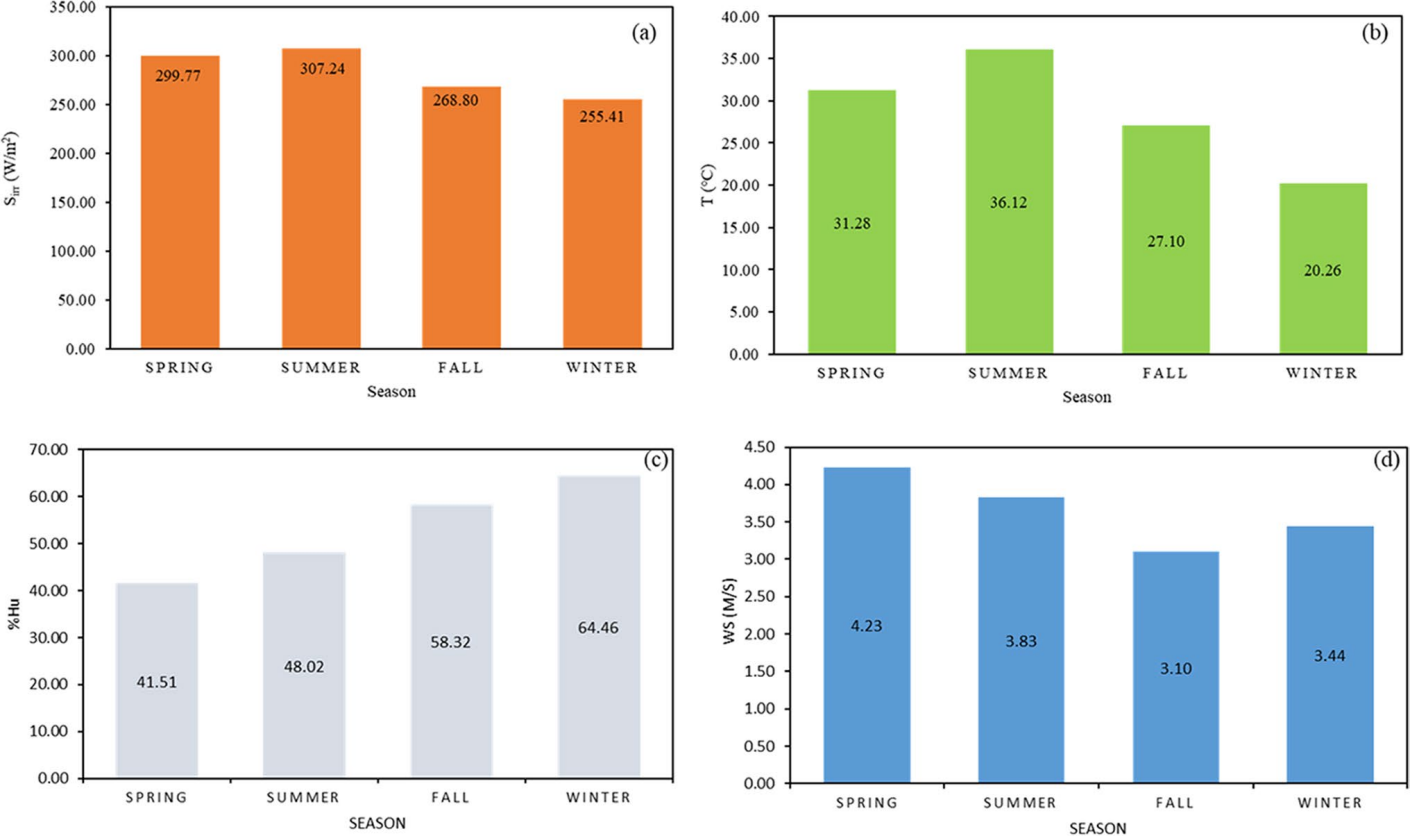
Seasonal average weather data for the entire year. Showing **a** S_irr_, **b** T, **c** %H_u_ T, and **d** W_s_

The average values of weather data in each season during the whole year were examined. Figure 5 presents the seasonal average weather data (S_irr_, %H_u_, T, and W_s_) for the studied area.

As could be seen in Fig. 5a, there is no big difference between the seasonal average S_irr_. The S_irr_ range from 255.41 to 307.24 W/m^2^. The highest S_irr_ value of 307.24 W/m^2^ occurs in summer, while the lowest value of 255.41 W/m^2^ occurs in winter. The distribution of the average T is shown in Fig. 5b. The seasonal T variation is not significant (ΔT_max_ ≈ 12 ^○^C). The highest average T value was observed in summer, which is equal to 36.12 °C, and the lowest average T in winter which is equal to 20.26 °C. The average %H_u_ is shown in Fig. 5c. The minimum value of %H_u_ occurs in the spring and then increases to reach a maximum value of 64.56 in the winter. It can be seen in Fig. 5d that the W_s_ is high in the spring season, with the highest average W_s_ value of 4.23 m/s.

### System characteristics

In this section, the characteristics of air inside the system such as temperature and velocity are illustrated. The impacts of these characteristics on P_elc_ production and water desalination were discussed. In addition, the relation between system components (PV, SC, and CT) and P_elc_ production and water desalination was discussed.

### Temperature profile

#### Results of CSCP

**Fig. 6:**
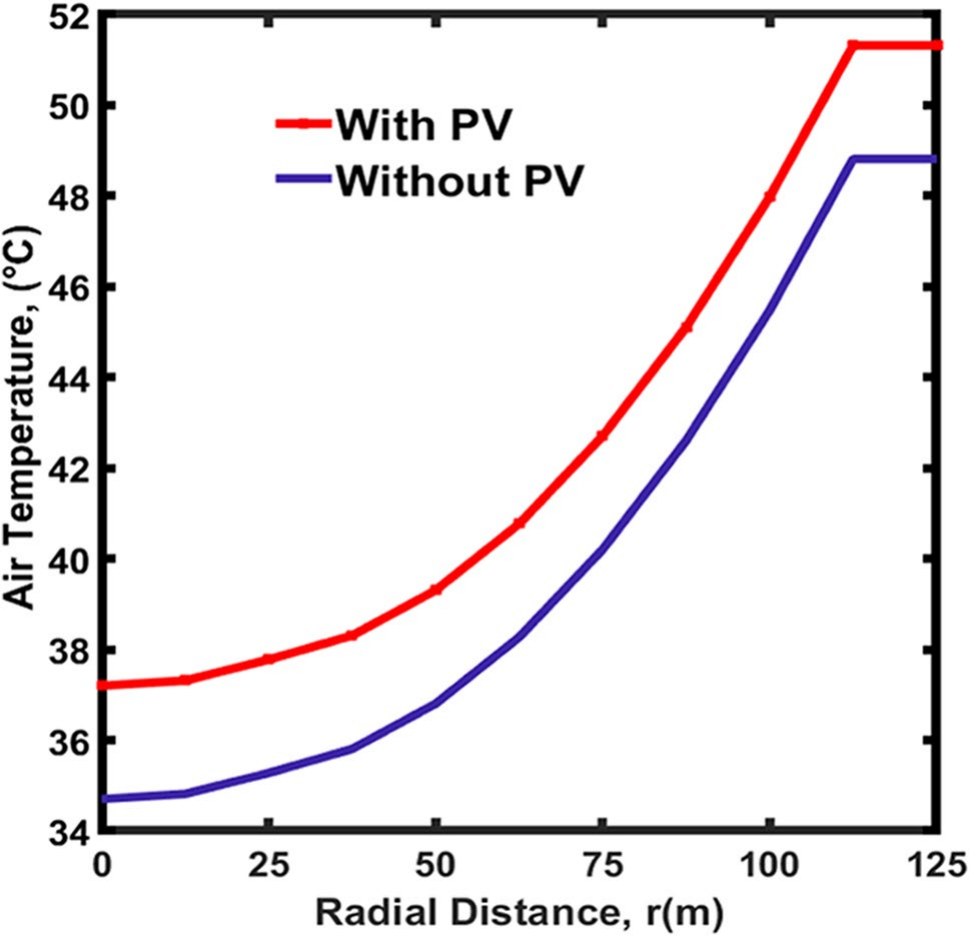
The temperature profile of the air under the collector with PV integrated and without PVPs

The profiles of the air temperature under the collector, i.e., from the system entrance, the radial distance of 0 m, to the center of the chimney, the radial distance of 125 m, are shown in Fig. 6. Generally, the temperature of the air is affected by the S_irr_ and the radius of the collector (r_c_). It was observed that as the S_irr_ and the radial distance increase, the temperature of the air under the collector increases. This is because the longer the distance the air travel under the collector, the more S_irr_ it is exposed to. The profile of the air temperature entering the CSC, without PV, is described in blue in Fig. 6, which was taken at sunrise time (6:00 am). The air temperature at the system entrance is equal to 35 °C, and then it increased as air moves toward the seawater pool and then flattens at approximately 48 °C over the water pool, at a radial distance of 110–125 m.

#### The impact of PVPs

The addition of the PVPs changes the air temperature profile as shown in red in Fig. 6. It was observed that adding PVPs increased the air temperature under the collector. The temperature at the entrance of the collector is heated up, and accordingly, the overall temperature profile is shifted up by approximately 3 °C. The temperature gradient within the structure would be directly affected by the 3 °C shift in the temperature profile. The 3 °C difference resulted in an increase in the temperature and pressure gradient within the structure, this change increased the velocity of the air inside the chimney and produced more dynamic power to operate the turbine and produce more P_elc_. The %H_u_ of the outside air also has a direct effect on the performance of the system. As the %H_u_ increases, the water evaporation decreases, leading to lower P_elc_. production. It is also worth indicating how the system’s performance is impacted by W_s_ and its hydrodynamics. Any potential changes in the wind’s direction and W_s_ could boost the kinetic energy of the air inside the chimney and improve the P_elc_. production. S_irr_ is another important factor to consider as it was observed that the P_elc_. production is based on intercepted solar energy. Tingzhen et al. (2008) showed the production of 35 kW under S_irr_ of 800 W/m^2^. Larbi et al. (2010) generated P_elc_ in the range 140 and 200 kW under S_irr_ and temperature range of 400 to 600 W/m^2^ and 20 to 38 °C, respectively.

### Air velocity profile

#### Results of CSC

**Fig. 7:**
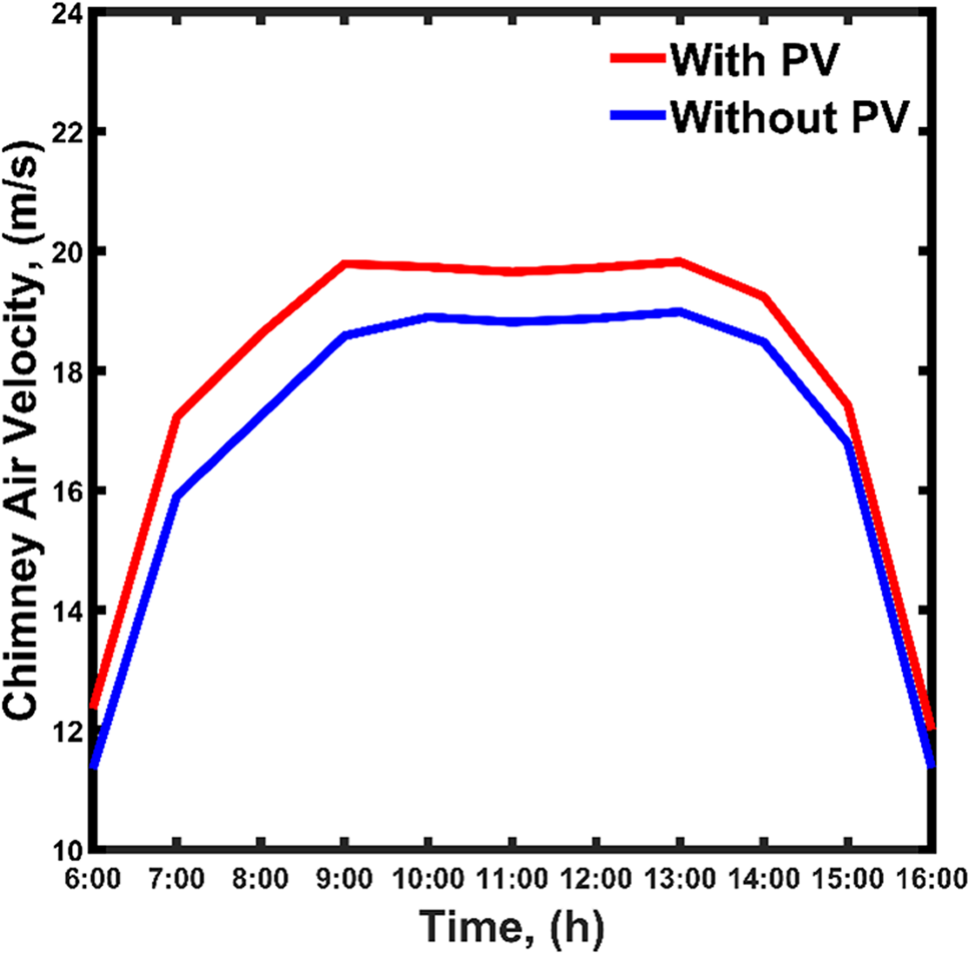
Velocity profile of the air exiting the chimney during the daytime with and without PVPs

The air velocity profile inside the chimney is described in Fig. 7. The velocity of the air entering the turbine plays an important role in electricity production. The air velocity was calculated as per Eq. (51) in the supplementary material. It was observed that the air velocity increases by increasing the ΔT between the air inside the chimney (i.e., at a radial distance of 125 m) and air before entering the collector (i.e., at a radial distance of 0 m). After the velocity profile was determined, the P_elc_ production was determined using Eq. (60). There was a direct correlation between the temperature of the air inside the structure and the P_elc_. As the air temperature inside the chimney increases, the air velocity increases leading to an increase in P_elc_ production. The chimney air velocity profile without PV is shown (in blue) in Fig. 7. The profile is taken at the time between sunrise (6:00) and sunset (16:00) because the operation of SC is effective during the day due to the presence of S_irr_. The air velocity inside the structure starts increasing from 11 m/s at 6:00 to reach maximum values of 18 m/s at noontime, then decreases and reaches back to 11 m/s.

#### Impact of PVPs

As mentioned before, adding PV to the system would create an additional heating effect that increases the air temperature inside the chimney. This will have a huge impact on developing high air velocity within the structure, and thus on the P_elc_ production. The chimney air velocity profile with PVPs is shown (in red) in Fig. 7. The impact of adding PVPs is significant, especially at noontime where the peak value is shifted up by approximately 2 m/s. Adding PVPs to the system has a secondary effect on increasing the chimney air velocity.

### Electricity production

The addition of PVPs to the SC has improved the system productivity compared with CSC operated only by regular weather conditions. When the PVPs were added to the system, the air temperature inside the chimney and consequently the air velocity was increased and thus the yearly performance of the P_elc_ was significantly increased. Sangi et al. (2011) shown that increasing S_irr_ enhanced air velocity at a constant radius. The S_irr_ creates a temperature difference between its inside and outside and develops a natural air draft that accelerates towards the turbine at the bottom of the chimney and generates P_elc_.

### Twenty-four-hour profile

#### Results of traditional SCPP

**Fig. 8:**
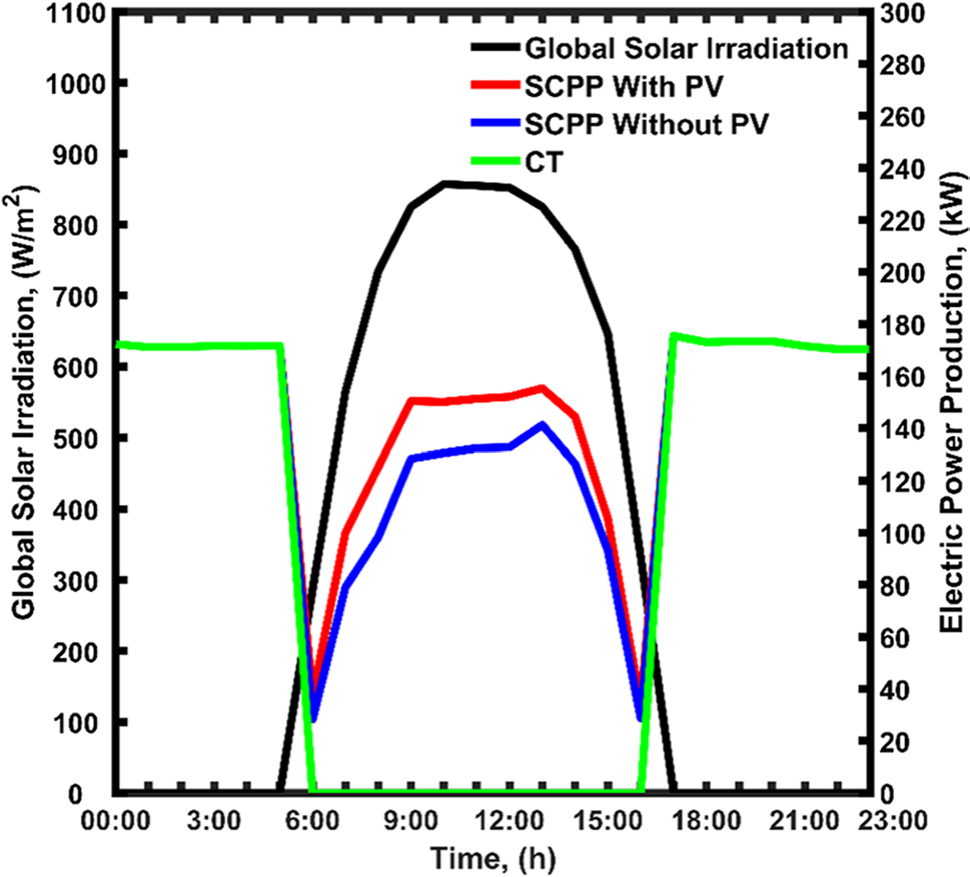
Twenty-four-hour profile of P_elc_ during the operation of the TRES as CTor SCPP with and without PV panel

Figure 8 presents the twenty-four profile of the P_elc_ production with and without PVPs on August 30th. The P_elc_ production of the CSCP is presented as a blue line in Fig. 8. The CSC is operational from sunrise (6:00) and sunset (16:00). The peak of P_elc_ production correlates with the peak of S_irr_, which is normally at noontime. Thus, the P_elc_ production increase from zero to reach the maximum of almost 140 kW. This production capacity is maintained between 9:00 and 15:00, and then slowly decreases to reach zero when there is no S_irr_.

The effect of adding PVPs to the system as previously mentioned was basically due to the increase in the air temperature and air velocity inside the chimney. The PVPs heating effect was translated into an increase in the P_elc_ production as shown in red in Fig. 8. The addition of PVPs has shifted the P_elc_ production from 140 kW to nearly 160 kW (with PV) all over the S_irr_ period. This increment in the P_elc_ production coincides with the improvement in chimney air velocity mentioned before in Fig. 7.

The operation of the structure as a CT is considered in the remaining time of the day, i.e., between sunset (16:00) and sunrise (6:00). The green line in Fig. 8 shows the P_elc_ production during the operation as CT. It was observed that the CT mode generates a fixed P_elc_ at approximately 170 kW. The obtained results show that the TRES structure offers an opportunity to generate sustainable P_elc_ all over the day (i.e., 24/7). The PV panel also has a major contribution to the total P_elc_ produced from the TRES structure. In addition to the power generation, the cooling duty achieved by the structure can be used by the nearby residential and industrial buildings for cooling, which adds up to the advantage of this structure. The proposed system has a small number of mechanical parts, except the turbine; therefore, the maintenance cost is expected to be very low.

The air velocity profile of the output air from the CSC reveals non-interacting boundary layers with local maxima near the center of the chimney. Similar trends were observed by Sakonidou et al. (2008). There was evidence that the high-pressure head and big difference between the inside and outside temperature contribute to the increase in the air velocity.

### Seasonal impact

**Fig. 9:**
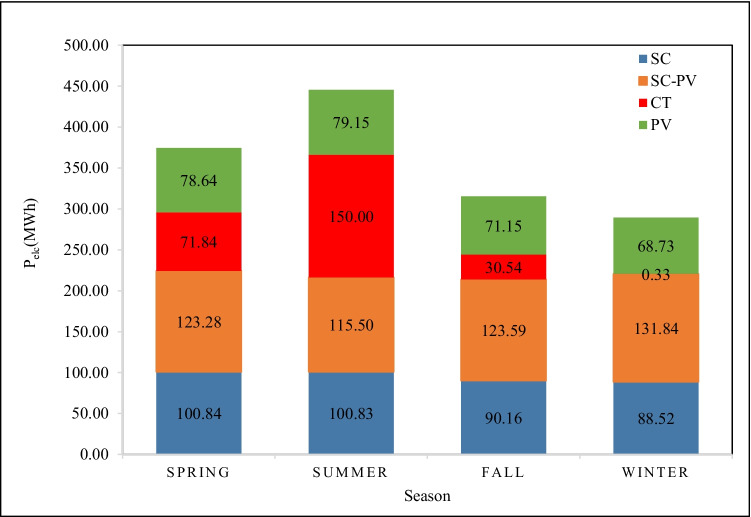
Seasonal profile of average P_elc_ from the CT, SCPP with or without PV

The amount of P_elc_ production of each system component, i.e., PVPs, SC, SC-PVPs, and CT, is relatively variable according to changes in weather conditions during the seasons. To have a better performance assessment, Fig. 9 shows the seasonal performance of each system component.

The operation of the TRES as CT produced P_elc_ in the range of 0.33 to 150.0 MWh. The highest P_elc_ production (150 MWh) was achieved in the summer, which is normally due to hot and dry weather. The lowest P_elc_ production (0.33 MWh) was observed to occur in the winter season due to high %H_u_ as discussed before. The performance of SC-PVPs is very close throughout the seasons, as it depends on S_irr_ with minor differences. The P_elc_ production from the PVPs, as expected, is the highest in the summer season (79.15 MWh), while the lowest production occurs in the winter (68.73 MWh). The SC has the best P_elc_ production in the winter season due to the big difference between the inner and outer air temperature.

### Yearly contribution

**Fig. 10:**
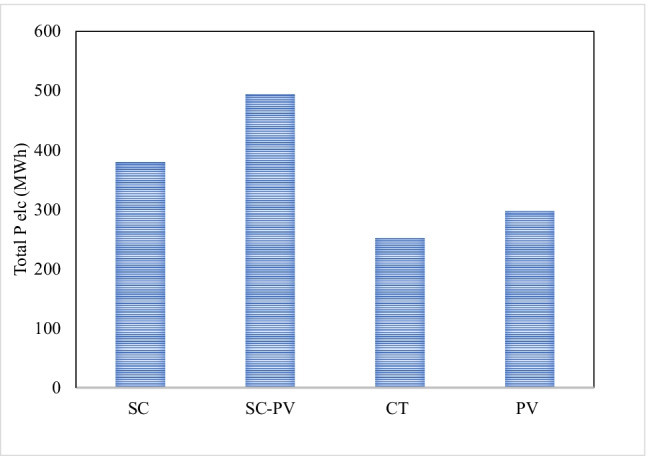
Yearly profile of average P_elc_ production from CT, SCPP with and without PV

The total yearly P_elc_ production by the TRES system was estimated to be 792 MWh as shown in Fig. 10. The SC-PV contributed to approximately 47% of the total P_elc_ production by producing 494 MWh. This is somewhat expected as the SC almost works in all the seasons. The CSC produced only 380 MWh as reported by Abdelsalam et al. (2021a). Therefore, combining the SC with PVPs (SC-PVPs) showed 2.1 folds improvement in P_elc_ production. Percentage-wise, the PVPs contributed to about 29% of the total annual P_elc_ production achieving 298 MWh. The CT contributed only 24% of the total P_elc_ production achieving 253 MWh. Although the contribution of the CT to the total P_elc_ production is not significant, it should be remembered that this P_elc_ was produced at night when there is no S_irr_ and the system is supposed to be not operational. In general, similar structures remain idle at night without any power production. Integrating the CT within the TRES boosts the electricity production by an additional factor of 0.66 when compared to the traditional SCPP. Integrating the PVPs adds another 0.784 factor of total power improvement. Hence, the overall improvement from 380 MWh to 1.044 GWh is 2.77 folds.

### Water production

#### Results of traditional SCPP

**Fig. 11:**
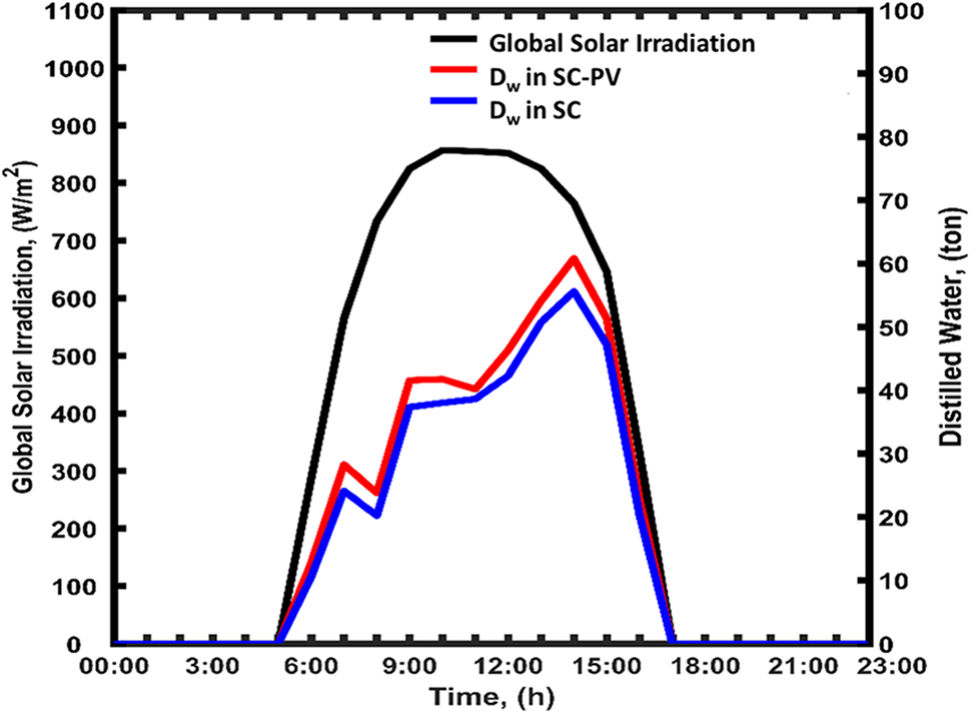
Twenty-four-hour profile of distilled water production in TRES with and without PVPs

Qatar has no fresh surface water resources. Hence, desalination, groundwater, and the reuse of treated sewage effluent are the country’s primary water resources. At least half of the country’s water comes from desalinated seawater. It is mainly used for municipal and industrial purposes, with roughly 99% of domestic water needs being met. Desalinated water production reached 533 MCM (Mega Cubic Meter) in 2015. In 2021, the total volume of desalinated water produced was 691 MCM. The TRES can be a solution to improve water production in this country. Figure 11 illustrates the distilled water production on August 30th from the TRES structure with and without PVPs. The distilled water production for the TRES system that has SC-PV was 1.13 to 1.30 higher than the conventional SCPP without PVPs. The results show that the distilled water production in the TRES starts from 06:00 to 18:00 with a maximum of 60 tons achieved at 14:00. During the same period, the maximum distilled water production for the TRES without PV was 55 tons. The is because  the convective heat effect of the PVPs heats the air and contributed to water evaporation. Not only does exchanging heat with the panels help to heat the air, but it may also improve the efficiency of the PVPs. The convective heat effect increase the temperature of the air and decrease its density. This hot air flows over the pool of seawater and increase water evaporation. The hot and humid air travels through the chimney column and its temperature decrease allowing the water to condenses at the walls of the chimney producing more desalinated water.

**Fig. 12:**
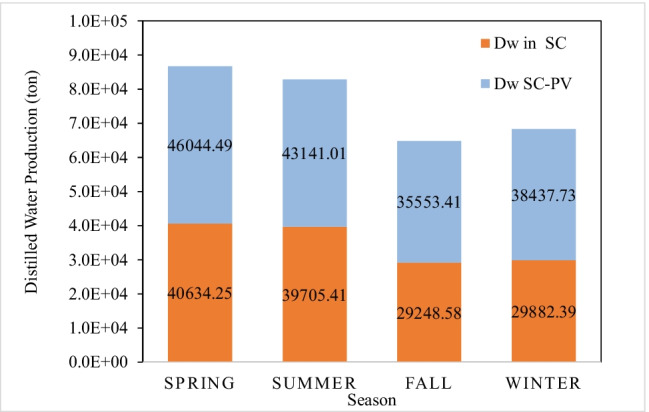
Seasonal profile of distilled water production

The seasonal productions of desalinated water from the SCPP with and without PV panels are presented in Fig. 12. The fall season marked the lowest desalinated water. The TRES without PVPs produced 29,248 tons, while SC-PVPs produced 35,553 tons. In the winter season, with low S_irr_, the system produced 29,882 tons or 35,437.7 tons for the structure without and with PVPs, respectively. The spring season marked the highest production of desalinated water (40,634.25 tons without PVPs and 46,044.49 tons with PVPs). In the summer season, the desalinated water production was 39,705.41 tons without PVPs and 43,141.01 tons with PVPs. Adding the results from all seasons showed that 163,142 tons of distilled water can be produced from the TRES compared to 139,443 tons produced by the conventional SCPP, marking a factor of 1.2 of improvement.

In summary, the proposed TRES system offers a technology that harnesses S_irr_ to produce P_elc_ and D_w_ all over the year. The proposed system is cost-effective as it has few mechanical parts and does not require frequent maintenance. It can be used in/off-grid communities and coupled with nearby industries to provide cooling utilities. The P_elc_ and D_w_ production are higher than the values reported by Niroomand and Amidpour (2013) and the results of an expensive wind supercharging SC as reported by Zuo et al. (2020). The is due to the contribution of the SC, PVPs, and CT in the system production. The obtained results also suggest that for better performance, the TRES could be improved by operating it with high-temperature gasses. Thus, integration with the power plant (PP) can develop a win–win situation where the PP uses the electricity and cooling utilities from the TRES system, while the TRES system can use the waste wheat to increase the P_elc_ and D_w_ production. Further research works are required to verify this fact. The buoyancy force of hot air can be converted to electrical energy in the TRES. As a result, excess heat from PP industries or even nuclear reactors can be used to raise the temperature of the air in the collector, contributing to the production of more P_elc_ and D_w_. Nonetheless, it should be always considered that heat from solar energy is free once the SCC is constructed and is not normally considered in the efficiency calculation. Fathi et al. (2018) showed through simulation work that connecting CSC to a 1000 MW nuclear power plant would increase the system thermal efficiency by 2-folds. This coupling strategy would lower investment costs while increasing revenue from power and water generation.

### System efficiency and environmental analysis

The addition of the PV panels and the CT boosted the P_elc_ production of the traditional SC. Hence, the annual, efficiency of the TRES was estimated using Eq. (1) and it was found to be 0.860% compared with only 0.313% for the traditional SCPP. The new system has 2.77 folds of P_elc_ production improvement.

The annual amount of the GHG emission reduction due to the use of the proposed TRES was calculated based on a conversion value of 0.95 kg CO_2_ eq./kWh and using Eq. (2). It was estimated that the new design can reduce the annual GHGs emission by 990 MT contributing to the calls for the protection of the environment.

1 $$\eta =\frac{{P}_{elc}}{\frac{1}{4 }\pi \left({D}_{col}^{2} -{ D}_{ch}^{2}\right) I}$$

2 $$Mass\;of\;{CO}_2\;\left(kg\right)=\frac{0.95CO2\;eq}{kWh}.\;Electrical\;production\;kW$$

## Conclusion

This work presented a novel renewable energy system based on integrating three technologies — CT, PVPs, and CSC. The goal of the work was to propose the system as an enhanced solar chimney power plant with improved efficiency. The results showed that the proposed integration improved the efficiency by a factor of 2.77. It was recommended to consider the proposed system as the new baseline for traditional solar chimney power plants. Future work will include investigating expanding the integration of the PVPs in other areas of the system, such as the seawater pool or the base. The TRES offers an outstanding opportunity to produce electricity and drinking water while reducing the annual GHGs emission by 990 MT and contributing to the calls for the protection of the environment. The annual P_elc_ and desalinated water production from the TRES system was found to be 792 MWh and 163,142 tons, achieving 2.1-folds and 1.2 higher than conventional SCPP. This integration might provide co-cooling for the panels, hence improving the efficiency of the PVPs and the solar chimney power plant. The potential of using the TRES system as a cooling utility required further investigation. The continuous operation of the TRES enhanced the utilization factor, reduce the dependence on fossil fuels, and therefore reduced CO_2_ emissions to the atmosphere. It is highly recommended to connect this design to an artificial intelligence algorithm to predict the performance under different weather conditions and control the mode of operation CT or SC to maximize output power.

### Supplementary Information

Below is the link to the electronic supplementary material.

Supplementary file1 (DOCX 206 KB)

## Data Availability

Data and materials are available to be shared upon request.
