# Supplementary material for: Triple-renewable energy system for electricity production and water desalination
Source: Environ Sci Pollut Res Int. 2022 Aug 29;30(44):98895–906. doi: 10.1007/s11356-022-22547-2 (PMC10533634; doi:10.1007/s11356-022-22547-2)
Supplement: Supplementary file 1 — Supplementary file1 (DOCX 206 KB) [file 11356_2022_22547_MOESM1_ESM.docx]

**Supplemental Material**

Detailed mathematical model.

**Nomenclature**

| A | Area: (m^2^) |
| --- | --- |
| H | Height, (m) |
| c_p_ | Specific heat capacity, (J/kg.K) |
| D | Diameter, (m) |
| I | Solar irradiation intensity, (W/m^2^) |
| Pelc | Electrical Out Power, (W) |
| T | Temperature, (K) |
| Q_out_ | The heat transfer between the chimney and the ambient, (W) |
| h | Heat transfer coefficient, (W/m^2^. K) |
| hfg | Latent heat of water evaporation, (W/m^2^. K) |
| $\bar{m}$ | Mass flow rate, (kg/sec) |
| $NOCT$ | Nominal Operating Cell Temperature (K) |
| g | Acceleration of gravity, (m/s^2^) |
| q | Heat transfer rate, (W/m^2^) |
| i | Enthalpy |
| r | Radius, (m) |
| k | Air thermal conductivity |
| dh | Hydraulic diameter, (m) |
| f | Friction factor |
| rw | Water Pool Radius |
| $\overline{Nu}$ | Nusselt number |
| Pr | Prandlt number |
| Re | Reynolds number |
| d_h_ | Hydraulic diameter, (m) |
| *f* | Friction factor |
| Sh_D_ | Sherwood number |
| Sc | Schmidt number |
| Z | Available wind data height |
| Z_R_ | The height at which the velocity needed to estimate |
| Zo | The roughness height of the glass |
| $V_{w}$ | Wind velocity (m/s) |
|  |  |
| **Greek Symbols** |  |
| α | Absorptivity |
| η | efficiency, % |
| τ | Transmissivity |
| ρ | density, kg/m^3^ |
| σ | Stefan- Boltzmann constant, W/m^2^. K^4^ |
| ω | Humidity ratio |
| ε | Emissivity |
| μ | Air dynamic viscosity |
|  |  |
| **Subscripts** |  |
| air | Airflow |
| abs | Absorber plate |
| c | Convective heat transfer |
| ch | Chimney |
| cd | Condensated water |
| col | Collector roof |
| e | Evaporation |
| gls | Glass cover or convective heat transfer |
| r | Radiative heat transfer |
| out | Outside |
| ent | Entrance |
| wtr | Water |
| sky | Sky |
| ted | PV Tedlar |

**
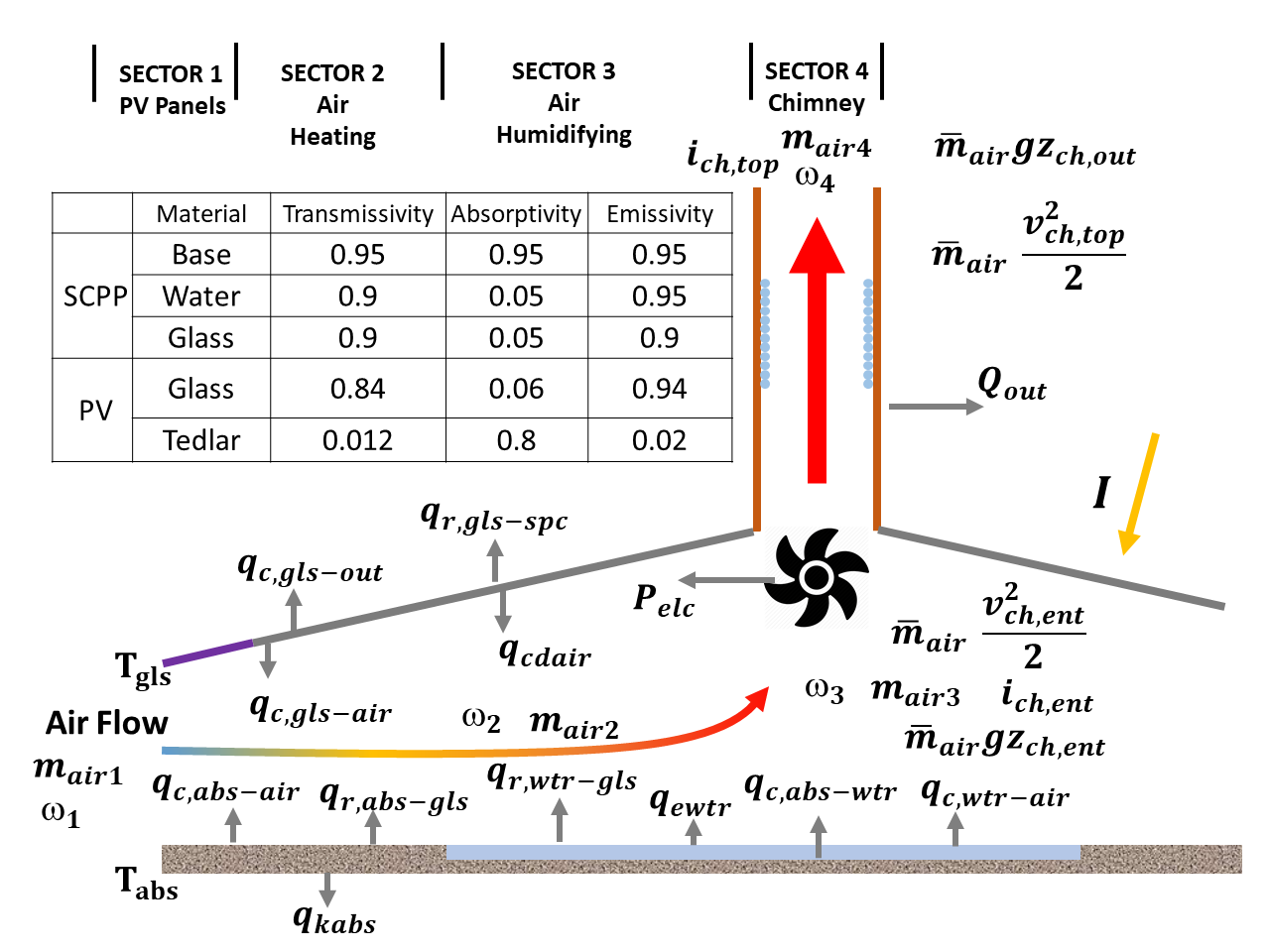
**

Figure 1S: detailed representation of the energy and material balance of the TRES

**Sector 1: PV Panels**

Using the equations below, the temperatures at each layer of the PV panels were calculated. The temperature of the PV glass cover was determined using Equation 1 [1]:

$M_{gls}C_{gls}\frac{dT_{gls}}{dt}=A_{m}\left[ \alpha_{gls}I+h_{r.sky-gls}\left( T_{sky}-T_{gls} \right)+h_{con,air}\left( T_{air}-T_{gls} \right)-h_{cnd.gls}\left( T_{gls}-T_{cell} \right) \right]$ (1)

Where:

$T_{gls}$ is the PV glass cover's temperature.

$T_{cell}$ is the PV cell temperature

$M_{gls}$is the mass of glass

$C_{gls}$ is the glass's thermal capacity

Equation 2 was used to get the equivalent temperature of the sky:

$T_{sky}=0.0552T_{air}^{1.5}$ (2)

Equations (3 and 4) were used to calculate the temperature of PV solar cells (in Kelvin and Celsius, respectively) [1]:

$T_{Cell_{k}}=T_{air}+\left( \left( NOCT-T_{air} \right)*\frac{I}{800} \right)$ (3)

$T_{Cell_{C}}=T_{Cell_{k}}-273$ (4)

The radiative rate of heat transfer between the PV panel’s transparent cover and the sky is determined by equation 5 [1]:

$h_{r,sky-gls}=\sigma\varepsilon_{gls}\frac{\left( T_{gls}^{2}-T_{sky}^{2} \right)\left( T_{cell_{k}}^{2}+T_{sky}^{2} \right)}{T_{gls}-T_{air}}$ (5)

Equation 6 define the wind-induced convective heat coefficient [1]:

$h_{con,air}=5.7+3.8V_{w}$ (6)

Equation 7 was used to calculate the temperature of the PV Tedlar layers [1]:

$M_{ted}C_{ted}\frac{dT_{ted}}{dt}=A_{m}\left[ \tau_{ted}\alpha_{ted}I\left( 1-\beta\right)+h_{cnd,t}\left( Tcell_{k}-T_{ted} \right)+h_{con,f-ted}\left( T_{ted}-T_{f} \right) -h_{c,air}\left( T_{ted}-T_{air} \right) \right]$ (7)

Where:

$T_{ted}$ is the PV Tedlar's temperature

$T_{f}$ is the air temperature under the PV panels

$h_{cnd,ted}$ is PV Tedlar's thermal conductivity

Equation 8 determines the radiative heat transfer coefficient between the cell and the tedlar of the PV panel [1]:

$h_{r,cell-ted}=\sigma\frac{\left( T_{cell}-T_{ted} \right)\left( T_{cell}^{2}+T_{ted}^{2} \right)}{\frac{1}{\varepsilon_{cell}}+\frac{1}{\varepsilon_{ted}}-1}$ (8)

**Sector 2:** **Solar Air Heating**

The energy balance equations for the air flowing in are as follows:

***Airflow:***

$q_{c,gls-air} + q_{c,abs-air}= - \frac{c_{p,air}\bar{m}_{air}}{2\pi r}\frac{dT_{air}}{dr}$ (9)

where $\omega_{1}= \omega_{2}$, from air mass balance equation

***Absorber:***

$q_{r,abs-gls}+{q_{c,abs-air} + q}_{kabs} = \alpha_{abs}\tau_{gls}I$ (10)

***Collector:***

$q_{c,gls-out}+q_{c,gls-air}{+ q}_{r,gls-spc}={\alpha_{gls}I + q}_{r,abs-gls}$ (11)

The convective heat transfer between the glass of the collector and the air flowing in the system is as follows:

$q_{c,gls-air}= h_{c,gls-air} (T_{gls}- T_{air})$ (12)

The convective heat transfer coefficient between the air under the collector and the glass is given according to the two following equations [2]:

$h_{c, gls-air}= \frac{0.2106+0.0026 V_{in} \left( \frac{T_{m}\rho_{air}}{\mu g(T_{gls}- T_{air})} \right)^{\frac{1}{3}}}{\left( \frac{\mu T_{m}}{(T_{gls}- T_{air})g{\rho_{air}}^{2}C_{P}k^{2}} \right)^{\frac{1}{3}}}$ (13)

Where, $T_{m}$ is the average temperature of $T_{gls}$ and $T_{air},\left[ T_{m}= \left( \frac{T_{gls}+T_{air}}{2} \right) \right]$.

$h_{c, gls-air}= \frac{\left( \frac{f}{8} \right)(Re-1000)Pr}{1-12.7\left( \frac{f}{8} \right)^{\frac{1}{2}}\left( {Pr}^{\frac{2}{3}}-1 \right)}\left( \frac{k}{d_{h}} \right)$ (14)

*When* $T_{air}> T_{gls}$*, then the value of* $h_{c, gls-air}$ *is based on the higher value produced from Eqs. (13) and Eqs. 146). However, if* $T_{gls}>T_{air}$*, Eqs. (14) is used.*

The convective heat transfer rate between the absorber and the air under the collector is as follows:

$q_{c,abs-air}= h_{c,abs-air} (T_{abs}- T_{air})$ (15)

The convective heat transfer coefficient between the air under the collector and the base is given according to the two following equations [2]:

$h_{c, abs-air}= \frac{0.2106+0.0026 V_{in} \left( \frac{T_{m}\rho_{air}}{\mu g(T_{abs}- T_{air})} \right)^{\frac{1}{3}}}{\left( \frac{\mu T_{m}}{(T_{abs}- T_{air})g{\rho_{air}}^{2}C_{P}k^{2}} \right)^{\frac{1}{3}}}$ (16)

Where $T_{m}$ is the mean temperature of $T_{abs}$ and $T_{air}$.

$h_{c, abs-air}= \frac{\left( \frac{f}{8} \right)(Re-1000)Pr}{1-12.7\left( \frac{f}{8} \right)^{\frac{1}{2}}\left( {Pr}^{\frac{2}{3}}-1 \right)}\left( \frac{k}{d_{h}} \right)$ (17)

*When* $T_{air}> T_{abs}$*, then the value of* $h_{c, abs-air}$ *is based on the higher value produced from Eqs. (16) and Eqs. (17). However, if* $T_{abs}>T_{air}$*, Eqs. (14) is used.*

The radiation heat transfer rate between the absorber and the solar collector is given as follows:

$q_{r,abs-gls}= h_{r,abs-gls} (T_{abs}- T_{gls})$ (18)

The radiative heat transfer coefficient is given follows [3]:

$h_{r, abs-gls}= \frac{\sigma\left( {T_{gls}}^{2}+{T_{abs}}^{2} \right)\left( T_{gls}+T_{abs} \right)}{\frac{1}{\varepsilon_{gls}}+\frac{1}{\varepsilon_{abs}}-1}$ (19)

Where $\sigma=5.67 \times{10}^{-8}\frac{w}{m^{2}k}$.

The convective heat transfer rate between the collector and the outside environment (sky) is as follows:

$q_{c,gls-sky}= h_{c,gls-sky} (T_{gls}- T_{sky})$ (20)

The convective heat transfer coefficient ($h_{c,gls-sky})$is given by [3]:

$h_{c,gls-sky}= 2.8+3.0v_{0}$ (21)

Where, $v_{0}$ is the wind speed above the horizontal glass of the collector.

The sky temperature is given by [4]:

$T_{sky}= T_{0}-6$ (22)

The radiation heat transfer rate between the collector and the sky is given as:

$q_{r,gls-sky}= h_{r,gls-sky} (T_{gls}- T_{sky})$ (23)

The heat transfer coefficient ($h_{r,gls-sky})$is given as follows [3]:

$h_{r,gls-sky}= {\sigma\varepsilon}_{gls} \left( \frac{{T_{gls}}^{4}-{T_{sky}}^{4}}{T_{gls}-T_{sky}} \right)$ (24)

**Sector 3: Air Humidification**

The energy balance equations for the seawter are as follows:$q_{c,abs-wtr}+ \alpha_{wtr}\tau_{gls}I= q_{ewtr}+q_{r,wtr-air}+q_{c,wtr-air}+c_{p,wtr}\bar{m}_{wtr}\frac{{dT}_{wtr}}{dt}$ (25)

We assume that that there is no spatial change the water temperature for the above equation.

Energy balance equation for air flow:

$q_{c,wtr-air}+q_{c,gls-air}= -\frac{c_{p,air}\bar{m}_{air}}{2\pi r}\frac{{dT}_{air}}{dr}$ (26)

Energy balance equation for the absorber:

$\alpha_{gls}\tau_{wtr}\tau_{gls}I=q_{c,abs-wtr}+q_{kabs}$ (27)

Energy balance equation for collector roof:

$q_{c,gls-air}+q_{r,wtr-gls}+\alpha_{gls}I=q_{r,gls-sky}+q_{c,gls-sky}$ (28)

The convictive heat transfer rate between the absorber and the seawater is as follows:

$q_{c,abs-wtr}= h_{c,abs-wtr} (T_{abs}- T_{wtr})$ (29)

Where, the heat transfer coefficient between the water and the base is given as:

$h_{c,abs-wtr}=135\frac{w}{m^{2}}$ [4] (30)

The evaporative heat transfer rate between the seawater and the air under the collector is as follows:

$\dot{q}_{ew}= \bar{m}_{ev} h_{fg}$ [5] (31)

The heat transfer coefficient ($\bar{m}_{ev})$is as follows [5]:

$\bar{m}_{ev}= h_{m}A_{ch}\Delta\rho$ (32)

Where, $\Delta\rho$ can be found from:

$\Delta\rho= \frac{\rho_{sat,Tairi}-\rho_{sat,Tairo}}{\ln\left( \frac{\rho_{sat,Twtr}-\rho_{sat,Tairo}}{\rho_{sat,Twtr}-\rho_{sat,Tairi}} \right)}$ (33)

$\rho_{sat,Tairo}= \rho_{sat,Twtr}+(\rho_{sat,Tairi}- \rho_{sat,Twtr})e^{-\frac{h_{m}\rho_{air}A_{c}}{\dot{m}_{air}}}$ (34)

To calculate the humidity ratio at the entrance of the chimney, the following can be used:

$w_{3}= \frac{\rho_{sat,Tairo}}{\rho_{air}}$ (35)

$h_{m}$ can be found using Sherwood number as follows:

${Sh}_{D}= \frac{h_{m}d_{h}}{D_{AB}}$ (36)

Where, $D_{AB}=0.26 \times{10}^{-4} \frac{m^{2}}{s}$.

${Sh}_{D}= \frac{\left( \frac{f}{8} \right)({Re}_{D}-1000)S_{c}}{1+12.7\left( \frac{f}{8} \right)^{\frac{1}{2}}\left( {S_{c}}^{\frac{2}{3}}-1 \right)}$ (37)

Given that $S_{c}= \frac{v}{D_{AB}}$.

${Nu}_{D}= \frac{\left( \frac{f}{8} \right)({Re}_{D}-1000)Pr}{1+12.7\left( \frac{f}{8} \right)^{\frac{1}{2}}\left( {Pr}^{\frac{2}{3}}-1 \right)}$ (38)

$\bar{{Nu}_{D}}= {Nu}_{D} \left( \frac{C}{\frac{L}{d_{h}}} \right)$ (39)

$f=\left( 0.79 lnRe-1.64 \right)^{-2}$ (40)

The radiation heat transfer rate between the water surface and the collector glass is as follows [4]:

$q_{r,wtr-gls}= h_{r,wtr-gls} (T_{wtr}- T_{gls})$ (41)

The heat transfer coefficient ($h_{r,wtr-gls})$can be found from:

$h_{r,wtr-gls}= \varepsilon_{eff}\sigma\left[ {T_{wtr}}^{2}+{T_{gls}}^{2} \right](T_{wtr}+ T_{gls})$ (42)

Where, $\varepsilon_{eff}$ is as follows:

$\varepsilon_{eff}=\left( \frac{1}{\varepsilon_{wtr}}+\frac{1}{\varepsilon_{gls}}-1 \right)^{-1}$ (43)

The convective heat transfer rate between the water and the air under the collector is as follows:

$q_{c,wtr-air}= h_{c,wtr-air} (T_{wtr}- T_{air})$ (44)

The heat transfer coefficient ($h_{c,wtr-air})$is as follows:

$h_{c,wtr-air}= \frac{0.2106+0.0026 V_{in} \left( \frac{T_{m}\rho_{air}}{\mu g(T_{wtr}- T_{air})} \right)^{\frac{1}{3}}}{\left( \frac{\mu T_{m}}{(T_{wtr}- T_{air})g{\rho_{air}}^{2}C_{P}k^{2}} \right)^{\frac{1}{3}}}$ (45)

Where, $T_{m}= \left( \frac{T_{wtr}+T_{air}}{2} \right)$.

*Humidification mass balance equation are as follows:*

$\frac{q_{ew}}{h_{fg}}= \bar{m}_{ev}= \bar{m}_{air}(w_{3}-w_{2})$ (46)

The density of the air entering the chimney is given as:

$\rho_{ent,ch}= \frac{\rho_{dair}(1+w_{3})}{(1+1.609w_{3})}$ (47)

**Sector 4: Solar Chimney**

The energy balance equation at the entrance (bottom) and exit (top) of the chimney is as follows:

$P_{elc}+Q_{out}= \bar{m}_{air}\left[ \left( \frac{v_{ch,ent}^{2}}{2}+gz_{ch,ent}+i_{ch,ent} \right)- \left( \frac{v_{ch,out}^{2}}{2}+gz_{ch,out}+i_{ch,out} \right) \right]$ (48)

Where $Q_{out}$ is the heat transfer between the chimney walls and the ambient outside the chimney.

The energy balance for the air inside the chimney is given by:

$Q_{out}=\bar{m}_{air}\left[ \left( i_{ch,ent}-i_{ch,out} \right)-\left( \omega_{4}-\omega_{3} \right)i_{wtr} \right]$ (49)

The mass balance equation for the air in and out of the chimney is as follows:

$\rho_{ch,ent}V_{ch}A_{in}= \rho_{out}V_{ch,out}A_{out}$ (50)

The enthalpy of the moist air entering the chimney is as follows:

$i_{ch,ent}= T_{a}+ w_{3} (2501.3+1.86T_{a})$ (51)

Where T_a_ is the air temperature at the inlet (in C^o^).

To calculate the mass flow rate of the water condensation, the following equation is used:

$\bar{m}_{wtr}=\bar{m}_{air}\left( \omega_{4}-\omega_{3} \right)$ (52)

To accurately calculate the heat transfer rate between the chimney and ambient, the chimney was vertically divided into equal sections. This is to take into consideration the change in outside wind velocity along the height of the chimney. The following equations were applied to each section of the chimney [6]:

$Q_{out}=hA\Delta T= hA (T_{air}- T_{o})$ (53)

$h= \bar{{Nu}_{D}}\frac{k}{D}$ (54)

$\bar{{Nu}_{D}}=C{Re}_{D}^{m} {Pr}^{n} \left( \frac{Pr}{Pr} \right)^{\frac{1}{4}}$ (55)

Where m = 0.6 and n = 0.37.

${Re}_{D}=\frac{V(Z_{R})D}{v}$ (56)

The wind velocity outside the chimney at different heights is estimated as follows [7]:

$V\left( Z_{R} \right)= V\left( Z \right)\frac{\ln\left( \frac{Z_{R}}{Z_{o}} \right)}{\ln\left( \frac{Z_{R}}{Z_{o}} \right)}$ (57)

The mass flow rate of the water condensation, can be calculate as follows:

$\bar{m}_{wtr}=\bar{m}_{air}\left( \omega_{4}-\omega_{3} \right)$ (58)

To calculate the velocity of the air as it enters the IC, the following equation was used [8]:

$V_{ch}=\sqrt{2gH_{ch}\frac{T_{ch,ent}-T_{out}}{T_{out}}}$ (59)

The output power produced by the turbine at the bottom of the IC was calculated as follows [8]:

$P_{elc}=\frac{1}{2}\rho_{en,ch}C_{f}A_{ch}V_{ch}^{3}$ (60)

where, $C_{f}$ is the turbine efficiency, set at 0.42.

**Cooling Tower**

Water sprinklers installed at the top of each CT channel, spray a mist of water in the air. The mist of water is absorbed almost immediately by the hot air to form cool air (vapor).

To calculate the enthalpy of the vapor the following was used:

$i_{vap}= i_{air} + {\omega_{vap}i}_{wtr}$ (61)

$i_{air}= c_{p,air} T_{out}$ (62)

The water enthalpy can be calculated as follows:

$i_{wtr}= c_{p,wtr} T_{wtr}$ (63)

The inlet enthalpy and exit enthalpy of the cooled air remains the same, because of this natural evaporation process. However, the temperature of the vapor decreases, due to the latent heat of vaporization. The change in the temperature can be calculated as follows:

$c_{p,air} T_{out}+(\omega_{air}2501.3+ T_{out}1.86)=c_{p,air} T_{vap}+ {(\omega}_{vap}2501.3+ T_{vap}1.86)$ (64)

To bring the water up to the sprinklers at the top of the cooling towers, water pumps are usually used to pump the water from a nearby reservoir. In the process of doing this, the water pumps consume some of the energy produced by the CT. The velocity of the air and power generated from the CT can be calculated from equations 59 and 60 respectively.

**References:**

[1] M. E. A. Slimani, M. Amirat, I. Kurucz, S. Bahria, A. Hamidat, and W. B. Chaouch, “A detailed thermal-electrical model of three photovoltaic/thermal (PV/T) hybrid air collectors and photovoltaic (PV) module: Comparative study under Algiers climatic conditions,” *Energy Convers. Manag.*, vol. 133, pp. 458–476, 2017, doi: 10.1016/j.enconman.2016.10.066.

[2] A. Islamuddin, H. H. Al-Kayiem, and S. I. Gilani, “Simulation of solar chimney power plant with an external heat source,” in *IOP Conference Series: Earth and Environmental Science*, 2013, vol. 16, no. 1, p. 12080.

[3] N. K. Bansal, J. Mathur, S. Mathur, and M. Jain, “Modeling of window-sized solar chimneys for ventilation,” *Build. Environ.*, vol. 40, no. 10, pp. 1302–1308, 2005.

[4] R. Kannan *et al.*, “Solar still with vapor adsorption basin: Performance analysis,” *Renew. energy*, vol. 62, pp. 258–264, 2014.

[5] N. A. S. Elminshawy, F. R. Siddiqui, and M. F. Addas, “Development of an active solar humidification-dehumidification (HDH) desalination system integrated with geothermal energy,” *Energy Convers. Manag.*, vol. 126, pp. 608–621, 2016.

[6] F. P. Incropera, A. S. Lavine, T. L. Bergman, and D. P. DeWitt, *Fundamentals of heat and mass transfer*. Wiley, 2007.

[7] S. Mathew, *Wind energy: fundamentals, resource analysis and economics*. Springer, 2006.

[8] L. Zuo, Y. Zheng, Z. Li, and Y. Sha, “Solar chimneys integrated with sea water desalination,” *Desalination*, vol. 276, no. 1–3, pp. 207–213, 2011, doi: 10.1016/j.desal.2011.03.052.
